# Effect of induced ruminal acidosis on blood variables in heifers

**DOI:** 10.1186/1746-6148-9-98

**Published:** 2013-05-06

**Authors:** Giorgio Marchesini, Roberta De Nardi, Matteo Gianesella, Anna-Lisa Stefani, Massimo Morgante, Antonio Barberio, Igino Andrighetto, Severino Segato

**Affiliations:** 1 Department of Animal Medicine, Production and Health, University of Padova, Legnaro, (PD) 35020, Italy; 2 Istituto Zooprofilattico Sperimentale delle Venezie, Legnaro, (PD) 35020, Italy

**Keywords:** Ruminal acidosis, Blood variables, Wireless rumen sensor, Heifers

## Abstract

**Background:**

Ruminal acidosis is responsible for the onset of different pathologies in dairy and feedlot cattle, but there are major difficulties in the diagnosis. This study modelled the data obtained from various blood variables to identify those that could indicate the severity of ruminal acidosis. Six heifers were fed three experimental rations throughout three periods. The diets were characterised by different starch levels: high starch (HS), medium starch (MS) and low starch, as the control diet (CT). Ruminal pH values were continuously measured using wireless sensors and compared with pH measurements obtained by rumenocentesis. Blood samples were analysed for complete blood count, biochemical profile, venous blood gas, blood lipopolysaccharide (LPS) and LPS-binding proteins (LBP).

**Results:**

The regression coefficient comparing the ruminal pH values, obtained using the two methods, was 0.56 (*P* = 0.040). Feeding the CT, MS and HS led to differences in the time spent below the 5.8, 5.5 and 5.0 pH thresholds and in several variables, including dry matter intake (7.7 vs. 6.9 vs. 5.1 kg/d; *P* = 0.002), ruminal nadir pH (5.69 vs. 5.47 vs. 5.44; *P* = 0.042), mean ruminal pH (6.50 vs. 6.34 vs. 6.31; *P* = 0.012), haemoglobin level (11.1 vs. 10.9 vs. 11.4 g/dL; *P* = 0.010), platelet count (506 vs. 481 vs. 601; *P* = 0.008), HCO_3_^-^ (31.8 vs. 31.3 vs. 30.6 mmol/L; *P* = 0.071) and LBP (5.9 vs. 9.5 vs. 10.5 μg/mL; *P* < 0.001). A canonical discriminant analysis (CDA) was used to classify the animals into four ruminal pH classes (normal, risk of acidosis, subacute ruminal acidosis and acute ruminal acidosis) using haemoglobin, mean platelet volume, β-hydroxybutyrate, glucose and reduced haemoglobin.

**Conclusions:**

Although additional studies are necessary to confirm the reliability of these discriminant functions, the use of plasma variables in a multifactorial model appeared to be useful for the evaluation of ruminal acidosis severity.

## Background

Ruminal acidosis is an ongoing problem in the dairy and feedlot sectors. It has been shown to cause consistent economic losses in dairy farming, primarily due to the reduction in milk yield and milk fat, premature culling and increased losses as a result of death [1]. In both the beef and dairy industries, many authors [2-5] have reported that ruminal acidosis is responsible for the onset of different pathologies, such as rumenitis, parakeratosis, metabolic acidosis, and laminitis. There are major challenges in improving the understanding of acute ruminal acidosis and subacute ruminal acidosis (SARA), including a wide range of responses observed under identical conditions [6] and difficulties in measuring the pH of the rumen, which require procedures such as rumenocentesis, oesophageal intubation or rumen cannulation.

There have been many attempts to use indirect variables to predict the ruminal pH based on symptoms or blood and metabolic indicators [2,7-9]. However, none of the variables alone have predicted the ruminal status of cattle, and only a few of the authors attempted to model metabolic variables to evaluate ruminal acidosis [10].

The aim of this study was to model the data obtained from the complete blood cell count, biochemical plasma profile, venous blood gas analysis, analysis of blood lipopolysaccharide (LPS) and LPS-binding proteins (LBP) to identify a subset of variables that could reliably indicate the severity of the induced ruminal acidosis in heifers.

## Results and discussion

### Animal health and body weight

Animal health was not compromised by the experiment as certified by a veterinarian at the end of each period. At the end of the trial, the heifers weighed an average of 382±17.3 kg with an average daily gain of 0.75±0.09 kg/d.

### Feed intake

**Figure 1:**
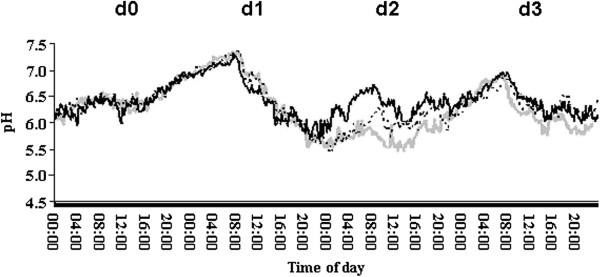
**Mean rumen pH trend according to the dietary treatment and the day.** CT = control, black continuous; MS = medium starch, black dashed; HS = high starch, grey continuous. d0 = feed restriction day (heifers fed CT diet at 0800 and 1200 h); d1 = challenge day (heifers fed CT, MS and HS diet at 0800, 1200 and 1800 h); d2 and d3 = first and second recovering days (heifers fed CT diet at 0800, 1200 and 1800 h).

**Figure 2:**
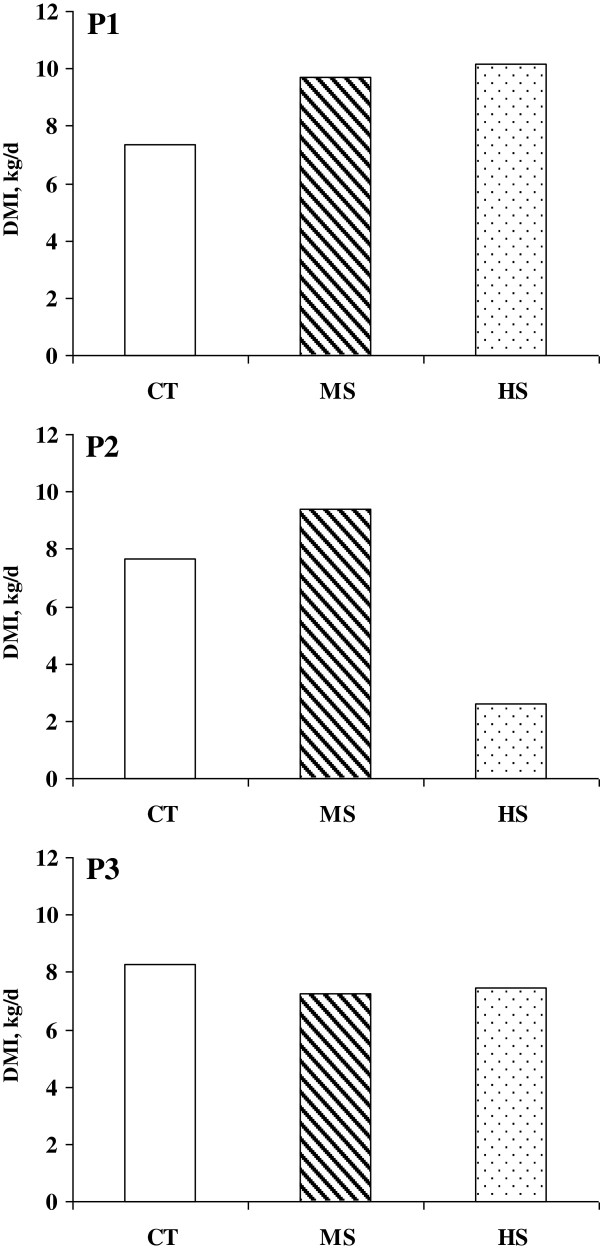
**Dry matter intake (DMI) on the challenge day (d1).** CT = control; MS = medium starch; HS = high starch. Treatment x Period: *P* = 0.030; SEM = 0.84. P1, P2, P3 = experimental periods.

**Figure 3:**
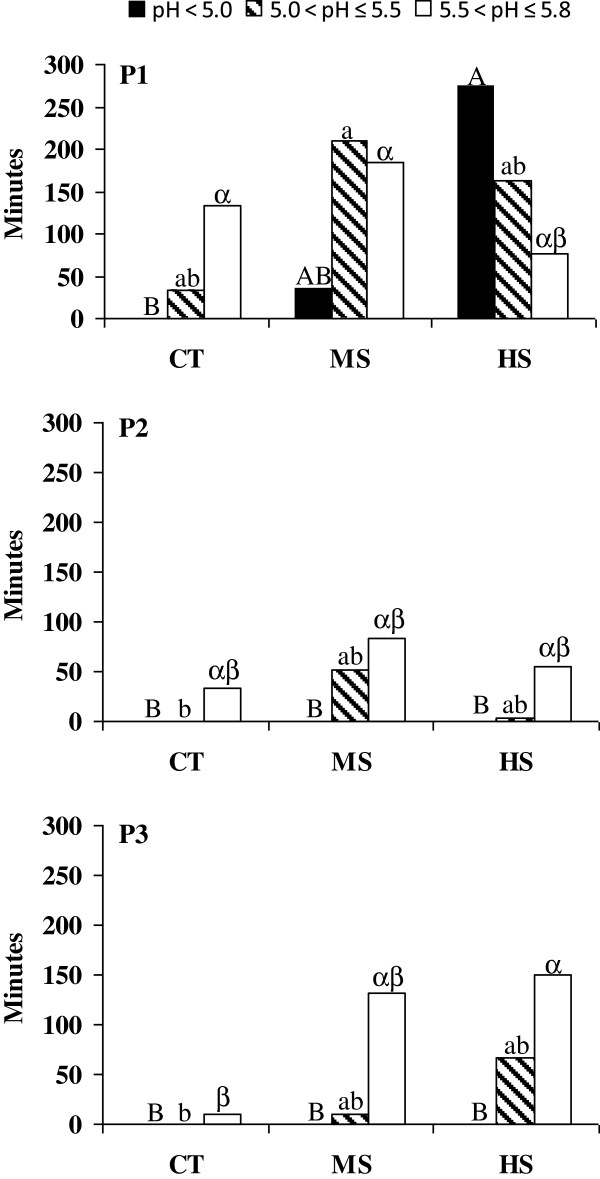
**Mean amount of time per day below the three ruminal pH thresholds.** Thresholds (pH < 5.0; 5.0 ≤ pH < 5.5 and 5.5 ≤ pH < 5.8) on d1 through d3, separated by the dietary treatment (CT = control, MS = medium starch, HS = high starch) and period (P1, P2, P3). Data are presented as averages and the associated *P* values are given by using the non parametric Kruskal-Wallis test. ^A, B^ Means of the amount of time per day below pH 5.0 with different superscripts are different (*P* < 0.05); ^a, b^ Means of the amount of time per day between pH 5.0 and 5.5 with different superscripts are different (*P* < 0.05); ^α, β^ Means of the amount of time between pH 5.5 and 5.8 with different superscripts are different (*P* < 0.05). The superscript letters refer to the interaction between the treatment and the period.

The dry matter intake (DMI) was significantly affected by the treatment, the day, the interactions period x day and treatment x period x day (Table 1). The interactions were significant because the challenge diets were provided only on the challenge day (d1) as specified in the protocol to induce acidosis. The lowest DMI was observed following the high starch (HS) treatment as a result of the ruminal pH drop (Figure 1) on the day after the d1 (d2) and it could be explained as an attempt to avoid the effects of the very low ruminal pH. Moreover, in the second period, the heifers that had experienced a pH below 5.0 after ingesting the MS diet in the first period dramatically reduced their intake with the HS diet (Figure 2). The reluctance to consume diets rich in starch after experiencing ruminal acidosis could be explained as a memory effect due to previously experienced ruminal acidosis despite the two-week recovery period. This result depends not only on the memory effect but also on individual sensitivity to ruminal acidosis. Heifers that consumed MS feed in the second period had less severe acidosis than the heifers that had fed on the same diet in the first period (Figure 3) and showed a lower reduction in the intake of HS feed in the third period (Figure 2).

**Table 1:** **Effects of dietary treatment (T, *n *= 18) and time period (P, *n *= 18) on DMI, ruminal pH, blood count, blood gas, haematological profile and acute phase proteins**

	**Treatment**^**1**^			**Period**^**2**^			***P*-value**			**SEM**
Trait	CT	MS	HS	1	2	3	T	P	T x P	
DMI, kg/d	7.7^a^	6.9^a^	5.1^b^	6.2	6.6	6.9	0.002	0.426	0.571	0.39
Nadir ruminal pH	5.69^a^	5.47^ab^	5.44^b^	5.23^b^	5.75^a^	5.62^a^	0.042	0.003	0.307	0.073
Mean ruminal pH	6.50^a^	6.34^b^	6.31^b^	6.15^b^	6.48^a^	6.52^a^	0.012	0.001	0.116	0.076
Max ruminal pH	7.13	7.09	7.08	6.97^b^	7.08^ab^	7.25^a^	0.423	0.001	0.054	0.090
Blood count and gas										
HGB, g/dL	11.1^ab^	10.9^b^	11.4^a^	11.7^a^	10.8^b^	10.9^b^	0.010	0.001	0.526	0.23
HCT, %	33.8^ab^	32.8^b^	34.1^a^	35.4^a^	32.3^b^	33.0^b^	0.027	<0.001	0.563	0.60
PLT, K/μL	506^b^	481^b^	601^a^	493	564	530	0.008	0.177	0.043	78.4
MPV, fl	4.2	4.1	3.9	4.0	4.2	4.0	0.542	0.840	0.266	0.26
pCO_2_, mmHg	52.0	50.3	50.4	50.4	50.9	51.4	0.126	0.502	0.137	0.63
pO_2_, mmHg	61.7	72.3	71.1	42.1^b^	71.4^ab^	91.6^a^	0.450	0.003	0.454	7.10
HCO_3_^-^, mmol/L	31.8^α^	31.3^αβ^	30.6^β^	31.8	31.3	30.7	0.071	0.127	0.081	0.45
O_2_Hb, %	87.6	87.9	86.3	77.5^b^	89.9^a^	94.5^a^	0.728	<0.001	0.683	2.01
RHb, %	9.9	9.8	12.4	20.4^a^	8.6^b^	3.1^b^	0.381	<0.001	0.721	1.96
sO_2_m, %	89.9	90.3	87.5	79.2^b^	91.4^a^	97.0^a^	0.393	<0.001	0.715	2.12
Haematological profile and acute phase proteins										
Glucose, mmol/L	4.34	4.37	4.32	4.42^α^	4.26^β^	4.35^αβ^	0.686	0.098	0.891	0.104
CHOL, mmol/L	3.52	3.37	3.45	3.27^b^	3.28^b^	3.79^a^	0.446	0.005	0.548	0.130
NEFA, meq/L	0.23	0.20	0.27	0.24	0.21	0.25	0.155	0.624	0.555	0.025
β-HB, mmol/L	0.28	0.31	0.29	0.26 ^β^	0.30^αβ^	0.31 ^α^	0.440	0.069	0.406	0.016
AST, U/L	78.2^ab^	72.1^b^	82.0^a^	76.1^αβ^	74.4^β^	81.7^α^	0.007	0.053	0.092	1.70
γGT, U/L	19.2	19.4	18.6	17.7^b^	19.2^ab^	20.4^a^	0.527	0.031	0.622	1.33
LBP, μg/ml	5.9^b^	9.5^a^	10.5^a^	10.5^a^	7.4^b^	7.9^b^	<0.001	0.014	0.221	0.92

^1^*CT* = control; *MS* = medium starch; *HS* = high starch.

^2^ Experimental periods.

^a–b^ Means within a row with different superscripts differ (*P* < 0.05). ^α– β^ Means within a row with different superscripts differ (*P* < 0.10).

HGB = haemoglobin; HCT = haematocrit; PLT = platelet count; MPV = mean platelet volume; pCO_2_ = partial pressure of carbon dioxide; pO_2_ = partial pressure of oxygen; HCO_3_^−^ = bicarbonate level; O_2_Hb = oxyhaemoglobin; RHb = reduced haemoglobin; sO_2_m = measured oxygen saturation; CHOL = cholesterol; β-HB = β-hydroxybutyrate; AST = aspartate aminotransferase; γGT = γ-glutamyl transferase; LBP = lipopolysaccharide-binding protein.

The day (D) effect (*P*) was as follows: DMI (< 0.001), mean pH (< 0.001), max pH (< 0.001), HGB (0.017), total protein (0.015), glucose (< 0.001), CHOL (0.014), NEFA (< 0.001), β-HB (0.012), AST (0.054) and LBP (0.012). The *P* value was > 0.05 for the other variables tested.

The interaction P x D effect (*P*) was as follows: DMI (0.011), mean pH (0.008), nadir pH (0.015), HGB (0.041), pO_2_ (0.049), glucose (0.001) and NEFA (0.026). The *P* value was > 0.05 for the other variables tested.

The interaction T x P x D effect (*P*) was as follows: DMI (0.008) and mean pH (0.047). The *P* value was > 0.05 for the other variables tested.

### Ruminal pH

The regression coefficient between the ruminal pH values obtained using sensors and rumenocentesis was 0.56 (*P* = 0.040), indicating a degree of agreement between the two methods.

The control (CT) treatment led to the highest nadir and mean ruminal pH values, whereas the lowest nadir pH level was reported for the HS treatment (Table 1). In the first period, the heifers showed the lowest values of maximum, mean and nadir pH (Table 1). The significant effects of the period and of the interactions treatment x period, period x day and treatment x period x day were due to the variations of DMI during the challenge day (Figure 2) and to individual ability to cope with the dietary factors that predispose animals to acidosis [6].

To evaluate the level of ruminal acidosis, the mean amount of time per day that the pH was below three ruminal pH thresholds (pH < 5.0; 5.0 ≤ pH <5.5 and 5.5 ≤ pH < 5.8) was determined and is reported in Figure 3. The ruminal pH fell below 5.0 during the first period for the HS and MS treatments when the animals experienced acute ruminal acidosis. The heifers feeding on MS during the first period had a pH between 5.0 and 5.5 for the longest period of time, while the pH never dropped below 5.5 in the heifers that were fed the CT treatment in the second and third periods. The pH varied between 5.5 and 5.8, ranging from at least 10 min per day on the CT diet during the third period to 180 min per day on the MS diet during the first period (Figure 3). The large differences in the amount of time the pH was below 5.0 between periods were related to the DMI (Figure 2), individual sensitivity to acidosis and a possible memory effect in the animals that had previously experienced acidosis.

### Blood analysis

Of the treatments, the heifers fed HS showed the highest concentrations of haemoglobin (HGB), haematocrit (HCT), platelet count (PLT) and aspartate aminotransferase (AST). The LBP was higher in the heifers fed HS and MS, and the bicarbonate (HCO_3_^-^) level was the lowest in HS. The concentrations of HGB, HCT, and PLT were higher in the heifers that had ruminal acidosis for longer periods of time due to high ruminal osmotic pressure, which pulls fluid from plasma into the rumen and concentrates the blood components [3,11]. PLT could be influenced by the onset of damage to the rumen mucosa as a result of acidosis, as reported by other authors [5].

The concentration of LPS in the peripheral blood plasma was below the assay detection limit of 0.1 EU/mL for all treatments. This result was likely due to the high clearance rate of the LPS in the Kupfer cells of the liver, which resulted in the absence of LPS in the peripheral blood and caused an inflammation cascade that led to the production of LBP [12].

The higher levels of LBP in the heifers fed MS and HS were due to the high starch intake and reduction in the ruminal pH. Some authors [9] reported that the early hours following grain engorgement are characterised by the rapid growth of Gram-negative bacteria, which undergo cell lysis and release LPS following a reduction in the ruminal pH. The translocation of LPS from the digestive tract to the bloodstream increases the LBP levels as a consequence of the systemic immune response [12]. The clearing of LPS in the liver could explain the slight increase in AST, which is a non-specific liver enzyme [13] that indicates liver alterations. The drop (*P* < 0.10) in HCO_3_^-^ level represents a mechanism to contrast the incoming of metabolic acidosis as a result of ruminal acidosis [14].

In this study, the period significantly affected the blood count, gas composition and the haematological profile (Table 1). The first period, which was characterised by increased amount of time below the established pH thresholds (Figure 3), led to an increase (*P* < 0.05) in HGB, HCT, reduced haemoglobin (RHb) and LBP and showed a slightly higher (*P* < 0.10) level of glucose. During the first period, there was a reduction (*P* < 0.05) in the partial pressure of oxygen (pO_2_), oxyhaemoglobin (O_2_Hb), measured oxygen saturation (sO_2_m), γ-glutamyl transferase (γGT) and a slight (*P* < 0.10) decrease in β-hydroxybutyrate (β-HB), whereas cholesterol (CHOL) was similar to the second period and AST showed an intermediate value.

The variations in RHb, pO_2_, O_2_Hb and sO_2_m reflected the effects of the cellular buffering system, which represents one of the mechanisms to maintain the blood pH within a physiological range as reported in humans [15]. During ruminal lactic acidosis, excess organic acids that accumulate in the rumen are absorbed into the bloodstream at the risk of overwhelming the bicarbonate buffering system [14]. When the blood pH begins to drop in response to decreased HCO_3_^-^ levels, there is a shift in the oxyhaemoglobin dissociation curve and the red blood cells release oxygen to the tissues more readily, which increases the RHb and reduces the O_2_Hb, pO_2_ and sO_2_m [15]. The slightly higher glucose level in the first period was a consequence of the increased DMI of HS and MS on d1 (Figure 2), which were rich in starch and led to a higher absorption of glucose in the small intestine. The low level of β-HB and cholesterol in the first period could be related to an altered energy status in the animals. As reported by other authors [16], the high level of glucose could have lowered the β-HB concentration while the variation in cholesterol levels could be linked to interactions between many factors, including the DMI and the ruminal pH [17].

The highest concentration of AST and γGT in the third period could be due to stress on the liver as a consequence of the considerable variations in dietary patterns during the experiment.

A canonical discriminant analysis (CDA) was applied to the four ruminal acidosis classes (such categorisation is based on the amount of time the pH is below the established pH thresholds). The CDA was characterised by two significant (Wilks’ *λ* = 0.282, *F* approx = 3.76, df1 = 15, df2 = 97, *P* < 0.0001) axes, which accounted for 60% and 38% of the existing variation. Among all the blood variables, HGB, mean platelet volume (MPV), β-HB, glucose and RHb contributed the most to the discriminant model (Table 2). Contrary to our expectations, LBP, an acute phase protein that was reported to increase during ruminal acidosis [9], was not included in the model even though it was higher in MS and HS compared with CT (Table 1). A possible reason is that LBP showed a different trend between d1 and d3 (7.0, 9.6 and 9.4 μg/ml for d1, d2 and d3, respectively, *P* = 0.012) compared to the pH trend, i.e., nadir pH (5.58, 5.40 and 5.62 for d1, d2 and d3, respectively, *P* = 0.084). The variables selected in the model explain the status of dehydration (HGB), the production of new platelets in the bone marrow, which are possibly due to lesions at the ruminal level (MPV), the energy status (β-HB and glucose) and the activation of the cellular buffering system to maintain the blood pH within a physiological range (RHb). Although single variables cannot predict the presence and severity of ruminal acidosis due to the considerable variation in the ability of an animal to cope with a carbohydrate challenge, evaluating specific combinations of blood variables that can highlight the ongoing processes of adaptation to the ruminal stress in the animal appears to be a promising approach in diagnosing and monitoring ruminal acidosis.

**Table 2:** Summary of the steps for the interactive forward mode (stepwise) for the CDA

	**Wilks’ *λ***	***P-*value**
HGB	0.715	0.005
MPV	0.505	<0.001
β-HB	0.420	<0.001
Glucose	0.347	<0.001
RHb	0.280	<0.001

*HGB* = haemoglobin; *MPV* = mean platelet volume; *β-HB* = β-hydroxybutyrate; *RHb* = reduced haemoglobin.

**Figure 4:**
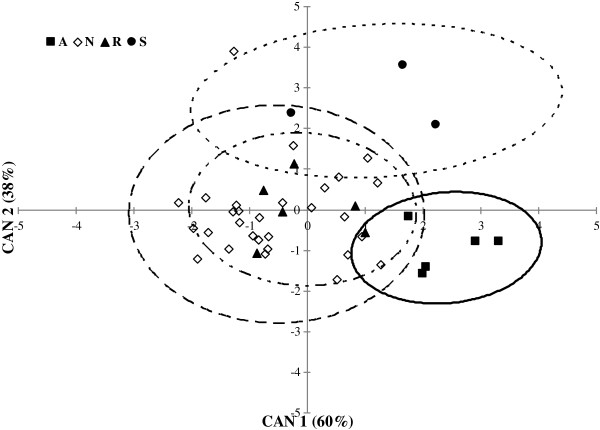
**Canonical discriminant analysis scattergram of the four classes of ruminal acidosis.** The axes (CAN 1 = 60% and CAN 2 = 38%) account for 98% of the total variability of the measured variables. Ninety-five per cent ellipses are drawn around each centroid of groupings. A = Acute ruminal acidosis (ellipse with a —— line); N = normal acidosis conditions (ellipse with —— line); R = Risk of SARA (ellipse with a - · - · - line); S = SARA (ellipse with a - - - - line). HS diet led to 4 episodes of acute ruminal acidosis in the first period, 1 episode of risk of acidosis in the second period and 1 episode of SARA in the third. MS diet caused 1 episode of acute acidosis, 2 episodes of risk of acidosis and SARA in the first period, whereas it led to 2 episodes of risk of acidosis in the third period. CT diet led to only 1 episode of risk of acidosis in the first period.

As reported in Figure 4, the scattergram relative to the total canonical structure expressing the correlation of HGB, MPV, β-HB, glucose and RHb with the canonical axes (CAN 1, *P* < 0.001 and CAN 2, *P* = 0.009) showed good separation between the different pH classes, with the exception of the animals classified as normal (N) or at risk of ruminal acidosis (R), which were not distinguished. Squared Mahalanobis distances (*D*^2^-Mahalanobis) obtained using CDA between the ruminal acidosis groups showed that acute ruminal acidosis (A) was different from the SARA (S) (*D*^2^-Mahalanobis = 4.9; *P* = 0.002), N (*D*^2^-Mahalanobis = 7.1; *P* < 0.001) and R groups (*D*^2^-Mahalanobis = 3.6; *P* = 0.010). SARA showed a significant separation from the R (*P* = 0.017) and N (*P* = 0.001) groups (*D*^2^-Mahalanobis = 3.2 and 5.2, respectively).

Although CAN 1 and CAN 2 represent the interactions among the five variables considered, according to the raw canonical coefficients (RCC), the separation between the ruminal acidosis classes along the CAN 1 axis, which was particularly evident between N and A, appeared to be strongly related to β-HB (RCC = 7.8) and glucose (RCC = 1.6). The difference in these variables between the animals experiencing acute ruminal acidosis compared with the others was associated with the higher energy status of the animals fed high grain diets. The separation between the acidosis classes along the CAN 2 axis, which was higher between the A and S heifers, appeared to be related to β-HB (RCC = 7.9) and MPV (RCC = 1.5), which could represent an increase in platelet formation due to the onset of ruminal lesions. Further research is needed to confirm this hypothesis.

## Conclusions

Many of the blood variables that were investigated showed significant differences between the three diets, although as few as five of them (HGB, MPV, β-HB, glucose and RHb) were sufficient to obtain a canonical structure (CDA). CDA appeared to significantly discriminate between the animals with a physiological ruminal status, SARA or acute ruminal acidosis. Despite these promising results regarding the use of plasma variables to evaluate the severity of short-term ruminal acidosis, additional studies are necessary to confirm the reliability of these discriminant functions during long periods of acidosis both in beef and dairy cattle.

## Methods

The experimental protocol was approved by the Animal Ethics Committee of the University of Padova, Italy (CEASA, approval number 88/2011) according to the national laws on the Ethics of Animal Experimentation.

### Animals and experimental design

Six crossbred Valdostana x Belgian Blue non-pregnant heifers with an average body weight (BW) of 334±14 kg were used. The animals were kept in loose housing conditions in an 88 square meter pen with a roof and natural ventilation equipped with six feeding stations and two waterers. The straw bedding was replaced daily, but not during the experimental periods to prevent the animals from feeding on it. The BW was measured at the beginning and the end of the trial. All of the heifers were examined at the beginning of each study period to evaluate their health status.

Before the beginning of the trial, the animals were allowed 15 days to adapt to the pen and the CT diet. Each experimental period lasted 5 days and was alternated with a rest period of two weeks during which the animals were fed a CT diet *ad libitum* and samples were not collected.

A 3 x 3 Latin square arrangement of treatments with 3-week experimental periods was used, and the heifers (*n*=6) were randomly assigned to the three dietary treatments according to the schedule reported in Table 3.

**Table 3:** Treatment sequence applied to the heifers throughout the periods

	**Treatment**^**1**^		
**Period**	**CT**	**MS**	**HS**
1	H1, H2	H3, H4	H5, H6
2	H5, H6	H1, H2	H3, H4
3	H3, H4	H5, H6	H1, H2

^1^*CT* = control; *MS* = medium starch; *HS* = high starch.

*H1-H6* = heifers used in the trial.

### Dietary treatments

Heifers were offered one of three diets characterised by the following different starch levels (Table 4): HS to induce acute ruminal acidosis, MS for SARA or low starch as CT. A similar acidosis challenge model was previously suggested by other authors [18].

**Table 4:** Formulation and composition of diets

	**Treatment**^**1**^		
**Item**	**CT**	**MS**	**HS**
Ingredients, % DM			
Permanent meadow 1^st^ crop	29.0	19.1	14.5
Dehydrated alfalfa hay	16.4	10.6	7.6
Soybean-based blend^2^	15.7	10.8	7.9
Dry beet pulp	6.3	4.3	3.2
Cereal mix^3^	25.6	16.1	13.0
Crushed linseed	4.5	3.1	2.0
Molasses	0.3	0.1	0.1
Vitamin and mineral mix	2.1	1.5	1.3
Maize meal (0.5 mm)	0.0	34.4	50.4
Diet Composition			
DM, %	89.1	87.6	87.8
Crude protein, % DM	16.4	14.3	13.2
Ether extract, % DM	4.5	4.2	4.2
Crude ash, % DM	8.7	6.2	5.0
NDF, % DM	33.0	26.3	20.9
Starch, % DM	17.3	33.4	42.8
Net energy for lactation, MJ/kg DM	6.91	7.66	8.00

^1^*CT* = control; *MS* = medium starch; *HS* = high starch.

^2^ 58% soybean meal and 42% extruded de-hulled soybean expeller.

^3^ 70% maize meal and 30% barley meal.

The animals were individually restricted and fed three times a day at 0800, 1200 and 1800 h. Water was continuously provided. At each meal, heifers were allowed to feed for approximately 1.5 h until each of the animals had stopped eating for at least 10 min. The residual feed was removed until the next meal. Heifers were fed 10 kg of their ration at each meal, and the feed that was not consumed was removed and weighed.

### Acidosis challenge model

Each experimental period was preceded by 3 baseline days (pre-challenge days d-3, -2 and −1) in which the heifers had access to the CT TMR three times per day. On the day before the challenge (restricted feeding day, d0), the feed was restricted to two meals (0800 and 1200 h) with a consequent reduction of DMI (2.8 kg on average). On d1, the HS, MS and CT diets were fed to induce acute acidosis or subacute acidosis or to maintain the physiological ruminal pH, respectively. On the following three days (d2, d3 and d4), all of the animals were fed the CT TMR three times per day.

### Feed intake and feed analyses

The weight of the feed offered and refused was recorded at each meal, and the total daily DMI was calculated as the sum of the amount ingested during the daily meals. The diets were sampled twice for each experimental week and analysed for chemical composition [19,20].

### Ruminal pH

The ruminal pH was continuously measured in all of the heifers during the entire trial using KB1001 wireless sensors (Kahne Limited, Auckland, New Zealand). Ruminal pH readings were collected every 10 min as suggested by other authors [21]. Fifteen days before commencement of the trial, the sensors were calibrated and delivered *per os* in the rumen using a sensor release device provided by the manufacturer. To verify the reliability of the pH values recorded by the sensors, ruminal fluid samples were collected from each heifer on the fourth day of each experimental period (d3) by rumenocentesis. The pH was immediately measured using a portable pH meter (Piccolo, Hanna Instruments, Villafranca Padovana, Italy) and compared with the values recorded by the sensors. Rumenocentesis was performed 4 hours after TMR distribution at 1200 h using a 13G, 105-mm needle [22,23].

The pH data from the sensors in each animal were summarised daily as the nadir pH, the maximum pH and the mean pH. The amount of time per day that the pH was below three ruminal pH thresholds (pH < 5.0; 5.0 ≤ pH < 5.5 and 5.5 ≤ pH < 5.8) was determined for each heifer during the three experimental periods. Although several rumen pH thresholds have been used to define acute ruminal acidosis and SARA [1,4,7,24], these threshold values were selected because pH < 5.0 leads to the destruction of both cellulolytic and lactate-using bacteria and protozoa and severely damages the rumen mucosa [5,25], pH < 5.5 is detrimental to the ruminal epithelium and VFA absorption [18,26] and pH < 5.8 is harmful to ruminal cellulolytic bacteria [18,27,28].

### Blood collection and analysis

Blood samples (20 mL) from the jugular vein were collected in lithium-heparin and K3 EDTA tubes (Vacuette, Greiner Bio-One, Kremsmuenster, Austria) from each animal at 0800 h on each experimental day immediately before the meal. The blood from the K3 EDTA tubes and one subsample of lithium-heparin-preserved blood was refrigerated (4°C) and analysed within 1 h for a complete blood cell count and blood gas analysis, respectively. The other subsamples were immediately centrifuged (1,500 g, 15 min, 4°C) for plasma separation and the plasma was preserved at −80°C until analysis.

The complete blood cell count with leukocyte formula was performed using an automated cell counter (Cell Dyn 3500, Abbott Laboratories, Abbott Park, Illinois, USA). Blood gas analysis was performed in a calibrated blood gas analyser (Synthesis 15, IL Instrumentation Laboratory SpA, Milano, Italy) to determine the following variables: pCO_2_, pO_2_, O_2_Hb and RHb. The HCO_3_^−^ level and sO_2_m were calculated. Measurements were performed as recommended by the National Committee of Blood Laboratory Standards [29]. The plasma was analysed for the following haematological variables: glucose, CHOL, non-esterified fatty acids (NEFA), β-HB, AST, γGT and LBP. With the exception of LBP, the haematological variables were measured with reagents supplied by Roche Diagnostics and Randox Laboratories Ltd. (NEFA and β-HB) for the Roche Cobas C501 automatic analyser (Roche Diagnostics, Indianapolis, IN, USA). The concentration of LPS in the plasma was determined by a chromogenic Limulus amoebocyte lysate (LAL) end-point assay (QCL-1000, Lonza Group Ltd. Basel, Switzerland) [30]. The plasma concentrations of LBP were measured [30] using a commercially available kit (HK503, HyCult Biotechnology, Uden, Netherlands). Samples were analysed in duplicate.

### Statistical analysis

The normality of the sample distribution was assessed using the Shapiro-Wilk test (PROC UNIVARIATE). The DMI, ruminal pH, blood gas analysis, plasma haematological profile and LBP data were analysed using a mixed procedure with a CS (compound symmetry) structure. The linear model is as follows:

$$\begin{array}{rll} Y_{\mathrm{ijkm}} & = & \mu +T_{i}+P_{j}+D_{k}+h_{l}+{\mathrm{TP}}_{\mathrm{ij}}+{\mathrm{TD}}_{\mathrm{ik}}+{\mathrm{PD}}_{\mathrm{jk}}\\ & & +{\mathrm{TPD}}_{\mathrm{ijk}}+\epsilon_{\mathrm{ijklm}} \end{array}$$

where μ is the overall mean; T_i_ is the fixed effect of the dietary treatment with 3 levels: CT, MS, and HS; P_j_ is the fixed effect of the period with 3 levels; D_k_ is the fixed effect of the day with three levels: d1, d2 and d3 (one, two and three days after the restricted feeding day); h_l_ is the random effect of the heifer (2 heifers *per* T_i_); TP_ij_, TD_ik_ and PD_jk_ are the interactions between the fixed effects; TPD_ijk_ is the interaction between the effects of the dietary treatments, period and day; and ϵ_ijklm_ is the random residual ~N (0, σ^2^_e_). Day was considered a repeated measure. If a significant F test was detected (*P* < 0.05), the treatment means (LS*means*) of the T_i_ and P_j_ were compared using the probability of differences (PDIFF) option and the Bonferroni adjustment test. The DMI data on d1 were also evaluated according to a linear random model that included the fixed effects of dietary treatment and period (repeated measures) along their interaction, the random effect of heifers and the random residual. Moreover, a regression coefficient between the rumenocentesis (covariate) and the boluses ruminal pH (data detected only in d3) was determined using the same mixed model.

The average amount of time for each heifer with a pH below the three established pH thresholds (pH < 5.0; 5.0 ≤ pH < 5.5 and 5.5 ≤ pH < 5.8) were not normally distributed (W-values < 0.90), even following transformation. These data were tested using the non-parametric Kruskal-Wallis criteria (PROC NPAR1WAY) to discriminate between the dietary treatments, periods and their interactions (PDIFF Bonferroni adjusted).

Stepwise (PROC STEPDISC) forward canonical discriminant analyses (CDA, PROC CANDISC) were performed separately on the plasma gas and metabolites data (independent variables) to discriminate between the four classes of ruminal acidosis (N, normal ruminal conditions, pH > 5.8; R, risk of ruminal acidosis, 5.5 < pH ≤ 5.8 and 5.0 < pH ≤ 5.5 for less than 4 h; S, subacute ruminal acidosis, 5.0 < pH ≤ 5.5 for at least 4 h; and A, acute ruminal acidosis, pH ≤ 5.0) [25,31,32]. The plasma variables that contributed the most to the discrimination of the ruminal acidosis classes were selected based on the F values (*P* < 0.15) as criterion for inclusion in the stepwise analyses. Wilks’ *λ* and the associated *F* approximation were used to test the significance and estimate the weight of each plasma variable in CDA. Based on the resulting plasma profile (5 variables reported in Table 3), the squared Mahalanobis distances were calculated to assess the proximity between the rumen acidosis statuses in the predefined classes.

All of the statistical analyses were performed using SAS software (2008; release 9.2).

## Competing interests

The authors declare that they have no competing interests.

## Authors’ contributions

GM, IA and MM designed the feeding trial which was conducted by RDN and MG, whereas AB and A-LS performed the chemical analyses. GM and SS analysed and interpreted the data, and drafted the article. All authors provide editorial content and have read and approved the final manuscript.

## References

[B1] Krause KM Oetzel GR Inducing subacute ruminal acidosis in lactating dairy cows J Dairy Sci 2005 88 3633 3639 10.3168/jds.S0022-0302(05)73048-4 16162537

[B2] Enemark JMD The monitoring, prevention and treatment of subacute ruminal acidosis (SARA): A review Vet J 2008 176 1 32 43 10.1016/j.tvjl.2007.12.021 18343172

[B3] Owens FN Secrist DS Hill WJ Gill DR Acidosis in cattle: A review J Anim Sci 1998 76 275 286 9464909 10.2527/1998.761275x

[B4] Plaizier JC Krause DO Gozho GN McBride BW Subacute ruminal acidosis in dairy cows: The physiological causes, incidence and consequences Vet J 2009 176 1 21 31 18329918 10.1016/j.tvjl.2007.12.016

[B5] Steele MA AlZahal O Hook SE Croom J McBride BW Ruminal acidosis and the rapid onset of ruminal parakeratosis in a mature dairy cow: A case report Acta Vet Scand 2009 51 1 39 10.1186/1751-0147-51-39 19840395 PMC2770524

[B6] Schwartzkopf-Genswein KS Beauchemin KA Gibb DJ Crews DH Hickman DD Streeter M McAllister TA Effect of bunk management on feeding behavior, ruminal acidosis and performance of feedlot cattle: A review J Anim Sci 2003 81 Suppl E149 E158

[B7] Enemark JMD Jørgensen RJ Kristensen NB An evaluation of parameters for the detection of subclinical rumen acidosis in dairy herds Vet Res Commun 2004 28 687 709 15609869 10.1023/b:verc.0000045949.31499.20

[B8] Gozho GN Plaizier JC Krause DO Kennedy AD Wittenberg KM Subacute ruminal acidosis induces ruminal lipopolysaccharide endotoxin release and triggers an inflammatory response J Dairy Sci 2005 88 1399 1403 10.3168/jds.S0022-0302(05)72807-1 15778308

[B9] Dong G Liu S Wu Y Lei C Zhou J Zhang S Diet-induced bacterial immunogens in the gastrointestinal tract of dairy cows: Impacts on immunity and metabolism Acta Vet Scand 2011 53 1 48 10.1186/1751-0147-53-48 21824438 PMC3161887

[B10] Brown MS Krehbiel CR Galyean ML Remmenga MD Peters JP Hibbard B Robinson J Moseley WM Evaluation of models of acute and subacute acidosis on dry matter intake, ruminal fermentation, blood chemistry, and endocrine profiles of beef steers J Anim Sci 2000 78 3155 3168 11132830 10.2527/2000.78123155x

[B11] Bernardini D Gerardi G Peli A Nanni Costa L Amadori M Segato S The effects of different environmental conditions on thermoregulation and clinical and hematological parameters in long-distance road-transported calves J Anim Sci 2012 90 4 1183 1191 10.2527/jas.2011-4113 22100587

[B12] Li S Khafipour E Krause DO Kroeker A Rodriguez-Lecompte JC Gozho GN Plaizier JC Effects of subacute ruminal acidosis challenges on fermentation and endotoxins in the rumen and hindgut of dairy cows J Dairy Sci 2012 95 294 303 10.3168/jds.2011-4447 22192209

[B13] Radostits OM Gay CC Hinchcliff KW Constable PD Veterinary Medicine: A Textbook of the Diseases of Cattle, Horses, Sheep, Pigs and Goats 2007 Philadelphia: Elsevier Health Sciences

[B14] González LA Manteca X Calsamiglia S Schwartzkopf-Genswein KS Ferret A Ruminal acidosis in feedlot cattle: Interplay between feed ingredients, rumen function and feeding behavior (a review) Anim Feed Sci Technol 2012 172 66 79 10.1016/j.anifeedsci.2011.12.009

[B15] Jones MB Basic interpretation of metabolic acidosis Crit Care Nurse 2010 30 5 63 69 10.4037/ccn2010521 20889514

[B16] Van Knegsel ATM Van Den Brand H Dijkstra J Tamminga S Kemp B Effect of dietary energy source on energy balance, production, metabolic disorders and reproduction in lactating dairy cattle Reprod Nutr Dev 2005 45 665 688 10.1051/rnd:2005059 16285910

[B17] Steele MA Vandervoort G AlZahal O Hook SE Matthews JC McBride BW Rumen epithelial adaptation to high-grain diets involves the coordinated regulation of genes involved in cholesterol homeostasis Physiol Genomics 2011 43 308 316 10.1152/physiolgenomics.00117.2010 21245418

[B18] Dohme F DeVries TJ Beauchemin KA Repeated ruminal acidosis challenges in lactating dairy cows at high and low risk for developing acidosis: Ruminal pH J Dairy Sci 2008 91 3554 3567 10.3168/jds.2008-1264 18765614

[B19] AOAC International Official methods of analysis, 17th ed. (2nd revision) 2003 Gaithersburg, MD, USA

[B20] Van Soest PJ Robertson JB Lewis BA Methods for dietary fiber, neutral detergent fiber, and nonstarch polysaccharides in relation to animal nutrition J Dairy Sci 1991 74 3583 3597 10.3168/jds.S0022-0302(91)78551-2 1660498

[B21] Palmonari A Stevenson DM Mertens DR Cruywagen CW Weimer PJ pH dynamics and bacterial community composition in the rumen of lactating dairy cows J Dairy Sci 2010 93 279 287 10.3168/jds.2009-2207 20059926

[B22] Gianesella M Morgante M Cannizzo C Stefani A Dalvit P Messina V Giudice E Subacute Ruminal Acidosis and Evaluation of Blood Gas Analysis in Dairy Cow Vet Med Int 2010 http://www.hindawi.com/journals/vmi/2010/392371.html 10.4061/2010/392371 PMC2952916 20953375

[B23] Morgante M Gianesella M Casella S Ravarotto L Stelletta C Giudice E Blood gas analyses, ruminal and blood pH, urine and faecal pH in dairy cows during subacute ruminal acidosis Comp Clin Path 2009 18 229 232 10.1007/s00580-008-0793-4

[B24] O’Grady L Doherty ML Mulligan FJ Subacute ruminal acidosis (SARA) in grazing Irish dairy cows Vet J 2008 176 44 49 10.1016/j.tvjl.2007.12.017 18328751

[B25] Nagaraja TG Titgemeyer EC Ruminal acidosis in beef cattle: The current microbiological and nutritional outlook J Dairy Sci 2007 90 Suppl E17 E38 17517750 10.3168/jds.2006-478

[B26] Gäbel G Aschenbach JR Müller F Transfer of energy substrates across the ruminal epithelium: Implications and limitations Anim Health Res Rev 2002 3 1 15 30 10.1079/AHRR200237 12400867

[B27] Russell JB Wilson DB Why are ruminal cellulolytic bacteria unable to digest cellulose at low pH? J Dairy Sci 1996 79 1503 1509 10.3168/jds.S0022-0302(96)76510-4 8880476

[B28] Mao S Zhang R Wang D Zhu W The diversity of the fecal bacterial community and its relationship with the concentration of volatile fatty acids in the feces during subacute rumen acidosis in dairy cows BMC Vet Res 2012 8 237 10.1186/1746-6148-8-237 23217205 PMC3582618

[B29] Moran R Considerations in the Simultaneous Measurement of Blood Gases, Electrolytes, and Related Analytes in Whole Blood: Proposed Guideline 1993 Miami: NCCLC

[B30] Khafipour E Krause DO Plaizier JC Alfalfa pellet-induced subacute ruminal acidosis in dairy cows increases bacterial endotoxin in the rumen without causing inflammation J Dairy Sci 2009 92 1712 1724 10.3168/jds.2008-1656 19307653

[B31] McLaughlin CL Thompson A Greenwood K Sherington J Bruce C Effect of acarbose on milk yield and composition in early-lactation dairy cattle fed a ration to induce subacute ruminal acidosis J Dairy Sci 2009 92 4481 4488 10.3168/jds.2008-1852 19700709

[B32] National Research Council Nutrient Requirements of Dairy Cattle 2001 7 Washington, DC, USA: National Academy Press

